# Comment on the article: “Prediction of the development of acute kidney injury following cardiac surgery by machine learning”

**DOI:** 10.1186/s13054-021-03798-w

**Published:** 2021-10-26

**Authors:** Hang Zhang, Yang Zhao, Tongtong Gu

**Affiliations:** 1 Department of Thoracic and Cardiovascular Surgery, Nanjing First Hospital, Nanjing Medical University, No. 68 Changle Road, Nanjing, 210006 China; 2 Department of Biostatistics, School of Public Health, Nanjing Medical University, No. 101 Longmian avenue, Nanjing, 211166 China; 3 Department of Clinical Pharmacy, Jinshan Hospital Affiliated To Fudan University, No. 1508 Longhang Road, Shanghai, 201508 China

## To the editor

We read with great interest the article published in *Critical Care* by Tseng et al. [1] reporting prediction of cardiac surgery-associated acute kidney injury (CSA-AKI) using machine learning (ML). Using SHapley Additive exPlanation (SHAP) analysis, the authors identified a set of intraoperative features attribute to AKI, highlighting the value of intraoperative data in AKI risk prediction.

Among 94 clinical features, intraoperative urine output (IUO) was identified as the most influential feature, which may reflect acute respond for renal function. However, a majority of patients (70.2%) received cardiopulmonary bypass (CPB). In the CPB setting, usually, IUO does not assure actual renal function as a result of cold diuresis and centrally shunted non-pulsatile blood flow [2, 3]. More importantly, intraoperative fluid balance was significantly different in patients with or without AKI (9.8 [7.7–12.8] vs. 11.8 [8.7–15.6] mL/kg/h, *P* < 0.001) and there was a great individual difference among two cohorts. The authors defined AKI based on the changes in postoperative serum creatinine (Scr) levels, while not adjusting the effect of fluid balance may significantly underestimate the incidence of AKI, as a positive perioperative fluid balance may dilute Scr levels [4]. Taken together, the association between IUO and AKI is complex and needs to be further evaluated after adjusting potential clinical confounders (e.g., intraoperative fluid infusion, conventional ultrafiltration, body temperature).

Typically, the benefits of ML start to become apparent when the sample size exceeds 5000–10,000. The authors validated the ML models in a relatively few patients cohort (202 patients and 49 AKI cases), with the area under the receiver operating characteristic curves (AUCs) ranging 0.781–0.843. In addition to AUC, more interpretable indicators should be introduced into the evaluation of a ML model such as accuracy, sensitivity, specificity, positive predictive value, negative predictive value, and F1 score, as adequate discrimination (AUC > 0.8) may not imply good performance in the sensitivity and positive predictive value of the model. In particular, in the serious clinical condition like CSA-AKI, sensitivity is emphasized over specificity [5]. These parameters can be easily obtained from a confusion matrix. Therefore, if the authors were able to calculate them, we believe that the models will be more credible and will present more practical value.

The rapid development of ML techniques will certainly facilitate the management of AKI patients. The authors made an important contribution in explainable ML technology (SHAP values). We look forward to more valuable researches on CSA-AKI using advanced ML algorithms.

## Data Availability

Not applicable.

## Abbreviations

CSA-AKI: Cardiac surgery-associated acute kidney injury
ML: Machine learning
IUO: Intraoperative urine output
Scr: Serum creatinine
AUC: Area under the receiver operating characteristic curve
